# Rod Microglia: A Morphological Definition

**DOI:** 10.1371/journal.pone.0097096

**Published:** 2014-05-15

**Authors:** Samuel E. Taylor, Cristina Morganti-Kossmann, Jonathan Lifshitz, Jenna M. Ziebell

**Affiliations:** 1 BARROW Neurological Institute at Phoenix Children's Hospital, Phoenix, Arizona, United States of America; 2 Department of Child Health, University of Arizona College of Medicine – Phoenix, Phoenix, Arizona, United States of America; 3 Department of Biology and Biochemistry, University of Bath, Bath, United Kingdom; 4 Phoenix VA Healthcare System, Phoenix, Arizona, United States of America; 5 Neuroscience Program, Department of Psychology, Arizona State University, Tempe, Arizona, United States of America; Massachusetts General Hospital and Harvard Medical School, United States of America

## Abstract

Brain microglial morphology relates to function, with ramified microglia surveying the micro-environment and amoeboid microglia engulfing debris. One subgroup of microglia, rod microglia, have been observed in a number of pathological conditions, however neither a function nor specific morphology has been defined. Historically, rod microglia have been described intermittently as cells with a sausage-shaped soma and long, thin processes, which align adjacent to neurons. More recently, our group has described rod microglia aligning end-to-end with one another to form trains adjacent to neuronal processes. Confusion in the literature regarding rod microglia arises from some reports referring to the sausage-shaped cell body, while ignoring the spatial distribution of processes. Here, we systematically define the morphological characteristics of rod microglia that form after diffuse brain injury in the rat, which differ morphologically from the spurious rod microglia found in uninjured sham. Rod microglia in the diffuse-injured rat brain show a ratio of 1.79±0.03 cell length∶cell width at day 1 post-injury, which increases to 3.35±0.05 at day 7, compared to sham (1.17±0.02). The soma length∶width differs only at day 7 post-injury (2.92±0.07 length∶width), compared to sham (2.49±0.05). Further analysis indicated that rod microglia may not elongate in cell length but rather narrow in cell width, and retract planar (side) processes. These morphological characteristics serve as a tool for distinguishing rod microglia from other morphologies. The function of rod microglia remains enigmatic; based on morphology we propose origins and functions for rod microglia after acute neurological insult, which may provide biomarkers or therapeutic targets.

## Introduction

Microglia contribute to the homeostasis of the central nervous system (CNS). Typically, four morphologically distinct microglia are reported: ramified, bushy (reactive), active, and amoeboid. In the non-pathological CNS, microglia have a small soma, little perinuclear cytoplasm, and a number of thin, branched processes that are covered in fine protrusions [1]. These ramified microglia constantly survey the microenvironment to readily respond against a pathological event. In the event of an insult, microglia rapidly change their morphology from ramified cells, to bushy reactive cells with thickened, retracted processes [2]–[4]. They migrate to the site of injury, proliferate, and participate in the presentation of antigens, phagocytosis of cellular debris and secretion of proteases, which promote microglia motility as well as extracellular matrix remodeling [1], [2]. If the damage is excessive, microglia continue to change their morphology with the number of processes declining and the cell body adopting an amoeboid shape, to the point where they become indistinguishable from blood-borne macrophages (Reviewed in [2]).

In addition to these well-defined types of microglia, a fifth less commonly reported population has been described: the rod microglia. These cells were first noted in 1899, when Nissl depicted a morphology distinct from today's well-known ramified and activated microglia. Rod microglia have a narrow cell soma and few planar processes, with polar processes that are entirely polarized. Historically, rod microglial cells have been associated with infections such as typhus, syphilis and sleeping sickness [5]. With improved sanitation and penicillin, these illnesses have declined and correspondingly investigation into the role rod microglia play in the CNS. Despite the initial descriptions more than a century ago, scant data exist on the role and morphological attributes of rod-microglia [6]–[13]. This may be explained, in part, by lack antibody markers to discriminate rod microglia from other morphologies of microglia.

The cellular architecture of specific brain regions can also influence microglia morphology [14], [15]. The most striking example is between microglia in grey versus white matter [14]. Microglia in white matter have a bipolar morphology, whereas those in the grey matter extend processes radially [14]. In an experimental model of medial forebrain bundle transection, rod microglia were observed along white matter tracts [6]. Following experimental stroke, rod microglia have been shown in the hippocampus [8]. Additionally, rod microglia have been observed in the hippocampus of experimental and clinical cases of epilepsy [7], [12]. Notably, all published images of rod microglia are in isolation, in contrast to the trains observed in traumatic brain injury [13] and laser-induced ocular hypertension [16]. No description of rod microglia function or morphological characteristics was given in any of these reports.

Moreover, activated microglia, described as rod-shaped or round-shaped, have been observed in neuronal degeneration in the brindled mouse, a model of genetic copper deficiency [9]. These cells were reported to be rod-shaped or round-shaped, however the accompanying pictures lacked clarity to clearly depict cellular features of these activated microglia. Typically publications that mention rod microglia have poor image resolution, further obscuring the characteristics of these cells. Both activated and rod microglia have also been described in human cases of subacute sclerosing panencephalitis (SSPE), Alzheimer's Disease and Wilson's Disease [11] and following bacterial injection into rodent brain [10]. In the latter study, double-labeling indicated that activated microglia wrapped axonal processes stained with the neurofilament marker; however the authors failed to mention whether or not these microglia had a rod-shaped morphology, and hypothesized that these cells are implicated with synaptic stripping.

Within the limited publications that mention rod microglia, most have poor image resolution, often at magnifications too low to appreciate cytoarchitecture. Furthermore, these publications omit the characteristics of these cells as well as excluding any speculations into the role of these cells in health and disease. Notably, in all published images rod microglia are depicted in isolation. In contrast, we recently observed rod microglia to form tightly organized trains along axons of cortical regions most vulnerable to damage in a model of diffuse TBI [13]. In this study, rod microglia were only associated with neuronal structures and not with glial cells, leading to our conclusion that these cells play a critical role in axonal damage and recovery after injury, rather than phagocytosis of cellular debris.

In order to more fully understand the role of rod microglia morphology, function and relationship to neuronal/axonal structures, we embarked on a morphological study whereby we measured the length, width and process number in the rat brain following diffuse TBI. These characteristics have never been systematically evaluated in either TBI or other neuropathologies. The features of rod microglia reported in this study offer the first clear description of these cells following a CNS insult, from which a pathophysiological function can be inferred.

## Materials and Methods

### Surgical Preparation & Diffuse Brain Injury Model

Experiments were conducted in accordance with NIH and approved by The University of Kentucky's animal care and use of laboratory animals (IACUC), with measures taken to minimize pain and discomfort. Adult male Sprague-Dawley rats, 350–375 g, were subjected to a midline fluid percussion injury (mFPI) consistent with previously described methods [13], [17]–[22]. Briefly, rats were anesthetized with 5% isoflurane in 100% O_2_ prior to the surgery and maintained at 2% isoflurane via nose cone. Rats were placed in a stereotaxic frame and a midline scalp incision was made, exposing the skull. A 4.8-mm circular craniotomy was performed (centered on the sagittal suture midway between bregma and lambda); with care not to disrupt the underlying dura or superior sagittal sinus. An injury hub was fabricated from the female portion of a Luer-Loc needle hub, which was cut, beveled, and scored to fit within the craniotomy. A skull screw was secured in a 1-mm hand-drilled hole into the right frontal bone. The injury hub was affixed over the craniotomy using cyanoacrylate gel and methyl-methacrylate (Hygenic Corp., Akron, OH) was applied around the injury hub and screw. The incision was sutured at the anterior and posterior edges and topical Lidocaine ointment was applied. Animals were returned to a warmed holding cage and monitored until ambulatory.

For the induction of the injury, surgically prepared rats were re-anesthetized with 5% isoflurane 60 to 90 min after surgery. The dura was inspected through the injury-hub assembly, which was then filled with physiological saline and attached to the male end of the fluid percussion device (Custom Design and Fabrication, Richmond, VA). Upon return of reflexive responses, a moderate injury (1.9–2.0 atm) was administered by releasing the pendulum onto the fluid-filled cylinder. Rats were monitored for the presence of a forearm fencing response and the return of the righting reflex as indicators of injury severity [19]. Sham animals were connected to the FPI device, but the pendulum was not released. The injury-hub assembly was removed *en bloc*, the integrity of the dura was observed, and bleeding was controlled prior to the incision being stapled. A moderate severity brain injury was determined by a righting reflex recovery time of 6–10 mins, sham-injured animals recovered within 15 seconds. Recovery was monitored post-operatively for three days, with no overt differences (e.g. weight, movement, grooming) observed between animals.

### Iba1 Immunohistochemistry

At designated time points (1, 2, 7 or 28 days; n = 3 per time point) post-injury or sham operation (n = 3), rats were overdosed with sodium pentobarbital (200 mg/kg i.p.) and transcardially perfused with phosphate buffered saline (PBS), followed by 4% paraformaldehyde in PBS. Following decapitation, the heads were stored in paraformaldehyde fixative solution containing 15% sucrose for 24 h, after which the brains were removed, placed in fresh fixative, and shipped for histological processing to Neuroscience Associates Inc. (Knoxville, TN). Rat brains were embedded into a single gelatin block (Multiblock Technology; Neuroscience Associates). Individual cryosections containing all the rat brains were mounted onto large glass slides. These slides were then immunostained for ionized calcium binding adaptor molecule 1 (Iba-1) to identify all microglia.

All image acquisition was conducted using an Olympus AX80 automatic research microscope with attached DP70 digital camera. The primary somatosensory barrel fields (S1BF) of the cortex were selected as the area of interest due to the prevalence of rod microglia in this region [13].

### Cell measurements and statistical analysis

Image analysis was conducted using NIH Image J software. Iba1 positive rod-microglia were identified in the S1BF of injured rats, these cells exhibited highly polarized processes. Firstly, the length and width of the soma was measured using the ‘straight line’ tool. The length was defined as the distance between the apical and basal tips of the soma (major axis); whereas the width was defined as the distance between the long edges (minor axis). Next, using the same tool, the length and width of the cell was determined. The cell boundary (including the soma and processes) was determined by the limits where the processes were too fine to be distinguished from the surrounding tissue.

To measure the primary branches, which were defined as a branch directly protruding from the soma, the ‘freehand line’ tool was used. The length of the primary branches for both the planes (long edges) and poles (apical and basal tips of the soma) were measured. The average lengths of both polar and planar branches were calculated for each individual rod microglial cell. Secondary branches were defined as those directly protruding from the primary branch. Tertiary branches and beyond were not quantified or measured.

For each brain-injured animal, 20 photomicrographs of rod microglia were measured in 3 separate brain sections for a total of 60 cells per animal, 180 cells per time point, and 720 cells for the study. For the sham-injured animals, observable rod microglia were fewer in number within the cortex; 10 photomicrographs were measured in 3 separate brain sections for a total of 30 cells per animal, and 90 cells for the study. Outliers were removed when they skewed the data from a normal distribution in the number of rod microglia (sham: n = 0; 1 d: n = 1 cell; 2 d: n = 5 cells; 7 d: n = 2 cells; 28 d: n = 3 cells); determined by D'Agostino & Pearson omnibus normality test (GraphPad Prism 5; La Jolla, CA). The data of each measurement was described by a box and whisker plot, whereby the mean, 25%, and 75% quartiles are used. We focus our analysis and interpretation on the means. To aide in representing the population distribution, frequency distribution histograms were also generated for all measurements and included below the appropriate figures. These graphs provide additional information regarding the frequency and distribution of the data, allowing the opportunity to observe any bimodal distribution in the data. The descriptive statistics are represented in the box and whisker plots, while the histograms show the spread and shape of the data set. One-way ANOVAs with a Tukey post-test were performed on the averages of the 180 measurements per post-injury time point and the 90 sham-injury controls using GraphPad Prism 5 for all normally distributed data. For data that was not normally distributed and ratio data, Kruskal-Wallis tests followed by a Dunn's multiple comparison tests were performed. All statistics are reported in the results section. P values less than 0.05 were considered statistically significant.

## Results

**Figure 1:**
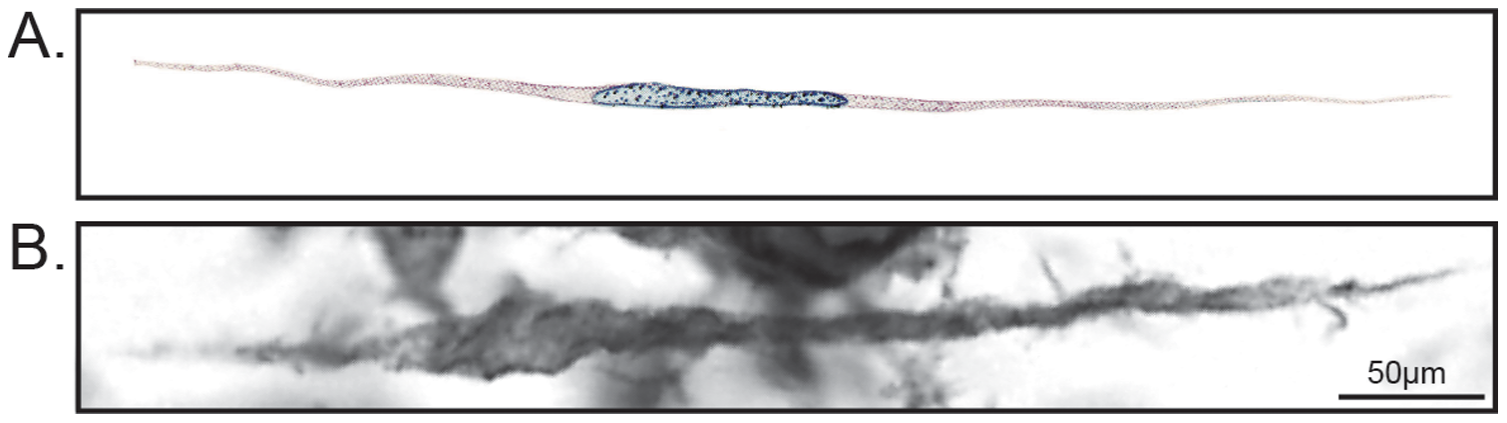
Rod microglia compared to Nissl's Stäbchenzellen illustration. A) Franz Nissl's illustration of Stäbchenzellen (rod cells) observed in patients suffering from syphilitic paralysis, as depicted by Spielmeyer [5]. B) Representative Iba1-positive rod microglia in the sensory cortex 7 days post-FPI, cell length∶cell width ratio of 12∶1. Note the similarities in the morphological characteristics between the two images.

Microglia with a rod-like cell body were observed in the cortex stained with Iba-1 of both sham-injured and diffuse brain-injured rats. In sham-injured animals, rod-like cells were sporadically found across the cortex and thalamus; however there was no appreciable alignment or polarization of these cells in any specific trajectory. After midline FPI, rod microglia were predominantly observed in the S1BF of the cortex, aligning perpendicular to the dural surface as previously reported [13], [17]. Rod microglia in brain-injured rats (Figure 1B) resembled those depicted by Franz Nissl in the late 19^th^ century in patients suffering from syphilitic paralysis (Figure 1A).

**Figure 2:**
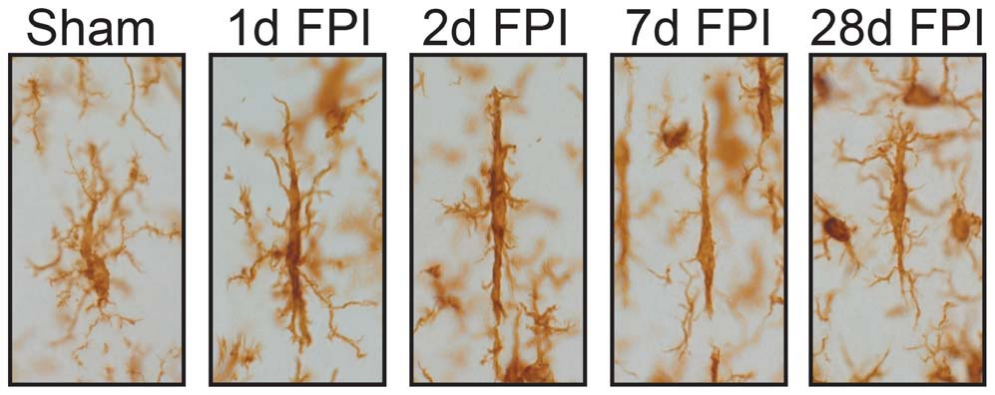
Rod microglia morphology and alignment post-FPI. Representative images of Iba1-positive microglia in the cortex of sham-injured and FPI rats. Sham-injured microglia showed a sausage-like cell body but no polarization of processes. However, as early as 1 day post-injury the retraction of planar processes was evident giving rise to rod morphology. Rod microglia continued to retract their planar processes at day 2 post-injury, and by day 7 there were few planar processes visible. By day 28, the cell body had begun to become more rounded and planar processes were returning.

The morphological characteristics of rod microglia measured in the histological analysis included soma length, soma width, cell length, cell width, as well as the number and length of the primary and secondary processes. These measurements were taken at day 1, 2, 7 and 28 post-injury, as well as from sham-injured brains. Although microglia in sham-injured rats displayed a sausage-like soma, they did not possess polarized processes. Visual changes in microglial morphology were observed as early as 1 day post-injury (Figure 2). Concordant to our previous study, the presence of rod microglia in the injured brain was noted at all time points post-injury that were investigated, with the highest numbers evident at day 7 [13]. The loss of processes and subsequent polarization were most evident at days 2 and 7 post-injury. Although microglia in sham rats displayed a sausage-like soma, there was a lack of polarized processes.

### Rod microglia narrow in response to diffuse brain injury

**Figure 3:**
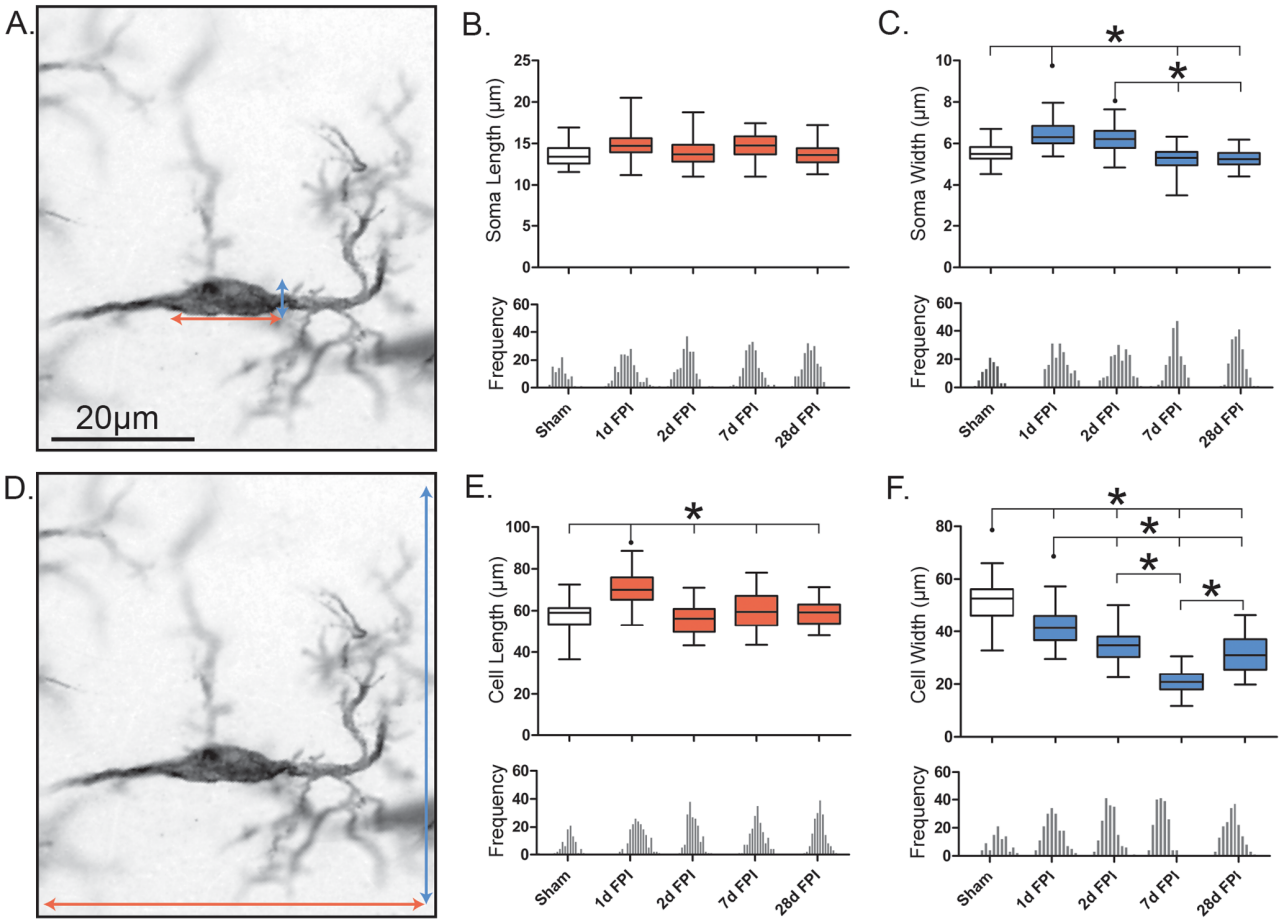
Retraction of planar processes gives the illusion of elongation. Morphological features of rod microglia cells and soma after diffuse brain injury. Rod microglia do not elongate, but rather narrow, post-injury. (A, D) Representative 28 day post-FPI Iba1-positive rod microglia, 100x. The orange and blue arrows illustrate the measured length and width respectively (µm) of the cell (A) and soma (D). The population of rod microglial cell length (B) and width (C) is described by a box and whisker plot, similarly for the soma length (E) and width (F). Notches on the x-axis of each frequency histogram represent the bin center for each specific time point, the bin center is determined from the total frequency histogram. In all cases a Gaussian distribution was observed. Significance was calculated using the average mean values for each time point (n = 3/time point; *, P<0.05, one-way ANOVA).

The soma of rod microglia was measured as the total visible size of the cell body, excluding the processes (Figure 3A). The length of rod microglia soma did not differ between sham and injured brains (F_(4, 14)_ = 3.39; p = 0.0534; Figure 3B). However, after FPI the soma width increased significantly (F_(4, 14)_ = 12.20; p = 0.0007; Figure 3C). The most striking change occurred at day 1 post-injury with a modest yet significant increase relative to sham and to all other time points after FPI (p<0.05). By day 7 post-injury, the soma width had returned to uninjured sham levels.

The cell length of rod microglia was measured based on the total visible size of the cell, including the soma and the processes (Figure 3D). In brain-injured rats, microglia cell length changed significantly (F_(4, 14)_ = 43.86; p<0.001; Figure 3E) to adopt a characteristic elongated rod morphology. At day 1 post-injury, the rod microglia cell length significantly increased over all other time points (p<0.001), returning to a cell length comparable to that of sham by day 2 post-injury and throughout the study period of 28 days. Although the width of rod microglia soma increased after injury, the width of the entire cell body plus processes was found to be significantly reduced at all time points post-injury as compared to the rod-like microglia in sham-injured rats (F_(4, 14)_ = 172.5; p<0.0001; Figure 3F). Moreover, the cell width continued to decrease significantly from 1 day through 7 day post-injury (p<0.001). It was only at 28 days post-FPI that cell width had begun to return to sham values.

To further distill the data we calculated the ratios of soma length∶soma width and cell length∶cell width (Table 1). The ratio of soma length∶soma width revealed a significant, albeit modest, increase in response to injury (H = 65.25, d.f. = 5, p<0.0001). Similar to the cell length∶cell width ratios, the change in soma length∶soma width was most evident on day 7 when compared to sham (p<0.001).

**Table 1:** Ratios of morphological data for rod microglia.

	Sham	1d FPI	2d FPI	7d FPI	28d FPI
**Cell Length∶Width**	1.17±0.02	1.79±0.03****	1.72±0.03****	3.35±0.05****	2.08±0.14****
**Soma Length∶Width**	2.49±0.05	2.41±0.05	2.33±0.05	2.92±0.07***	2.66±0.05
**Number of Primary Polar∶Planar Branches**	0.75±0.04	0.57±0.03***	0.65±0.03	0.72±0.03	0.65±0.03*
**Number of Secondary Polar∶Planar Branches**	0.93±0.08	1.40±0.10***	1.33±0.09***	2.13±0.18***	1.67±0.11***
**Ave Length Polar∶Planar Branches**	1.42±0.07	2.33±0.08***	2.13±0.08***	4.14±0.22***	2.94±0.15***
**Total Length Polar∶Planar Branches**	1.07±0.10	1.32±0.08	1.48±0.12	3.17±0.29***	1.89±0.17***

To further distill the changes observed in microglia morphology post-injury, ratios of the cell length∶width and soma length∶width analyzed. These data indicated that the most dynamic changes were occurring with the microglial processes, which were also analyzed as ratios of number of polar∶planar branches, average and total length of polar∶planar branches.

All significance is time post-injury compared to sham; *p<0.05, ***p<0.001 and ****p<0.0001.

The ratio of cell length∶cell width indicated the most striking and prolonged change of microglia morphology after injury (H = 170.8, df = 5, p<0.0001). These ratios were in fact increased significantly at all time points when compared to sham (p<0.001), with the largest increase on day 7, with almost a 3-fold increase in length compared to width of the cell. Quantitation of these ratios indicated a dynamic narrowing of the cell. These quantitative data suggest that the overall morphology of rod microglia is derived from the retraction of planar microglial processes. In order to determine more specific changes, we further analyzed the morphology of rod microglial processes post-injury.

### Retraction of rod microglial primary processes post-injury

**Figure 4:**
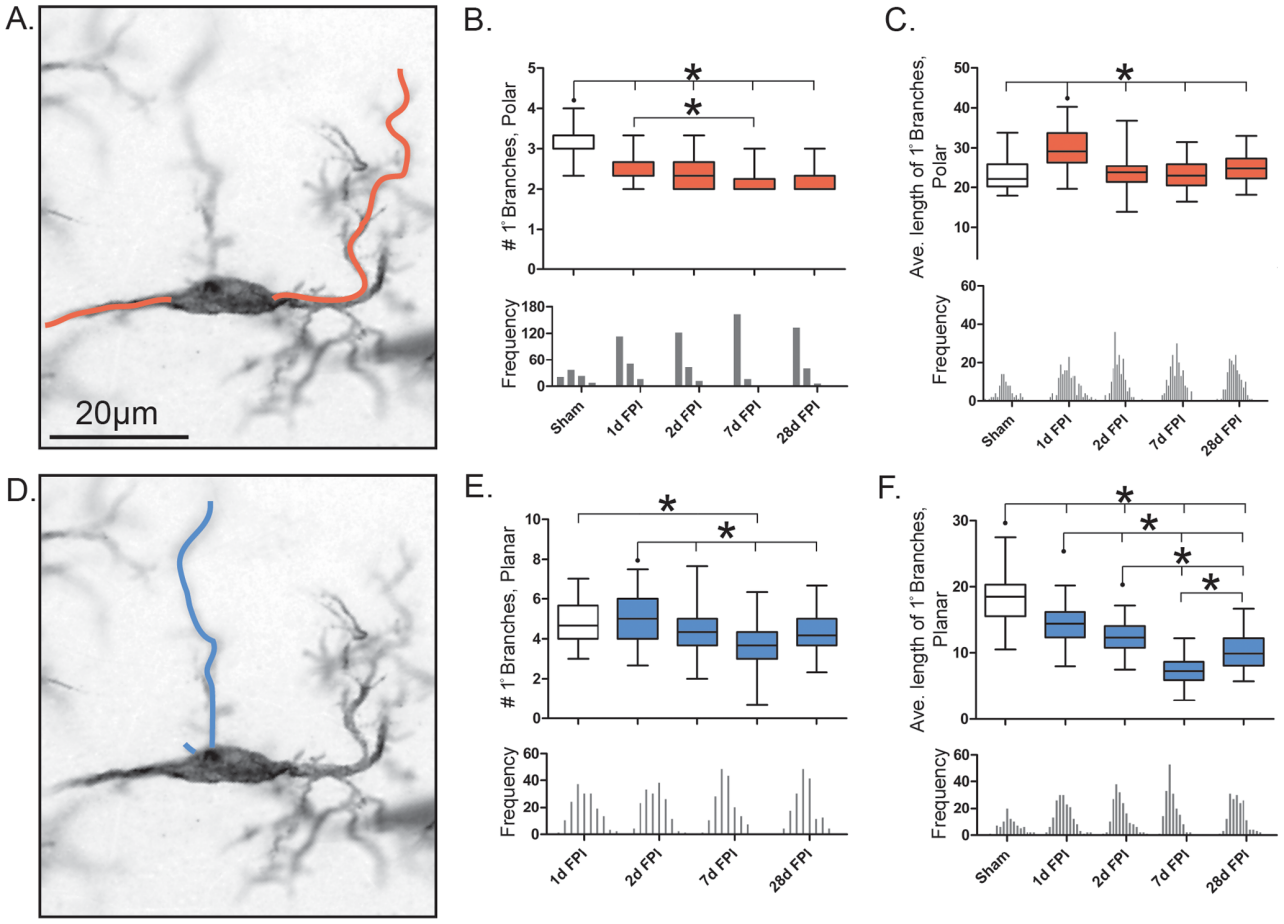
Retraction of rod microglial branches post-injury. (A, D) representative 28 day post-FPI Iba1-positive rod microglia, 100x. The orange and blue lines illustrate the measured primary polar and planar branches respectively. (B, E) Number of primary branches present on cell, polar and planar respectively. (C, F) Average length of the primary branches present on the cell (µm), polar and planar respectively. Populations described by a box and whisker plot. Notches of each frequency histogram represent the bin center for that specific time point. Unlike the average length of primary polar branches, the number of primary polar branches is heavily weighted to the left of the mean (negatively skewed). All other histograms show a Gaussian distribution. Significance was calculated using the mean values for each time point (n = 3/time point; *, P<0.05, one-way ANOVA).

The number of primary polar branches significantly decreased following injury (F_(4, 14)_ = 51.12; p<0.0001; Figure 4B), with a further reduction at 7 days post-injury from 1 day post-injury (p<0.05). The average length of the primary polar branches (µm) also changed significantly with injury (F_(4, 14)_ = 41.97; p<0.0001; Figure 4C). After a significant increase at 1 day post-injury compared to sham, the average length of the primary polar branches (µm) remained unchanged from sham at all proceeding time points (p<0.001). These measurements indicate an initial increase in the length of fewer polar processes, which return towards uninjured levels at later time points.

The number of primary planar branches changed significantly following injury (F_(4, 14)_ = 13.29; p<0.005; Figure 4E). This was most evident at day 7 post-injury, when compared to day 1 (p<0.001). The average length of the primary planar branches (µm) significantly decreased with injury compared to sham (F_(4, 14)_ = 140.3; p<0.0001; Figure 4F). A significant reduction of branch length was detected at all time points post-injury relative to sham (p<0.001). Again, the most striking decrease was at day 7 post-injury, where the length was significantly reduced compared to all other time points (p<0.001). These measurements indicate the elimination in number and decline in length of planar processes over time post-injury.

For a more global description of changes occurring in microglial process number and length post-injury, we examined the ratio of the number of primary polar branches∶number of primary planar branches as well as the length of the primary polar branches∶length of primary planar processes. The ratio of the average number of primary polar branches∶primary planar branches was altered with injury (H = 27.71, df = 5, p<0.0001; Table 1). These changes were most evident at day 1 post-injury (p<0.001, compared to sham), when the number of polar branches was 57% of the number of planar branches.

The ratio of average primary polar branch length∶primary planar branch length changed with injury (H = 211.5, df = 5, p<0.0001). These changes were evident at all time points post-injury when the polar length increased 1.5 to 3-fold from sham (p<0.001).

Taken together, these data suggest that the observed polarization of rod microglial cells is not due to the elongation of the primary processes but rather the retraction of planar processes and the narrowing of both the cell and soma.

### Decrease in the number of rod microglial secondary branches post-injury

**Figure 5:**
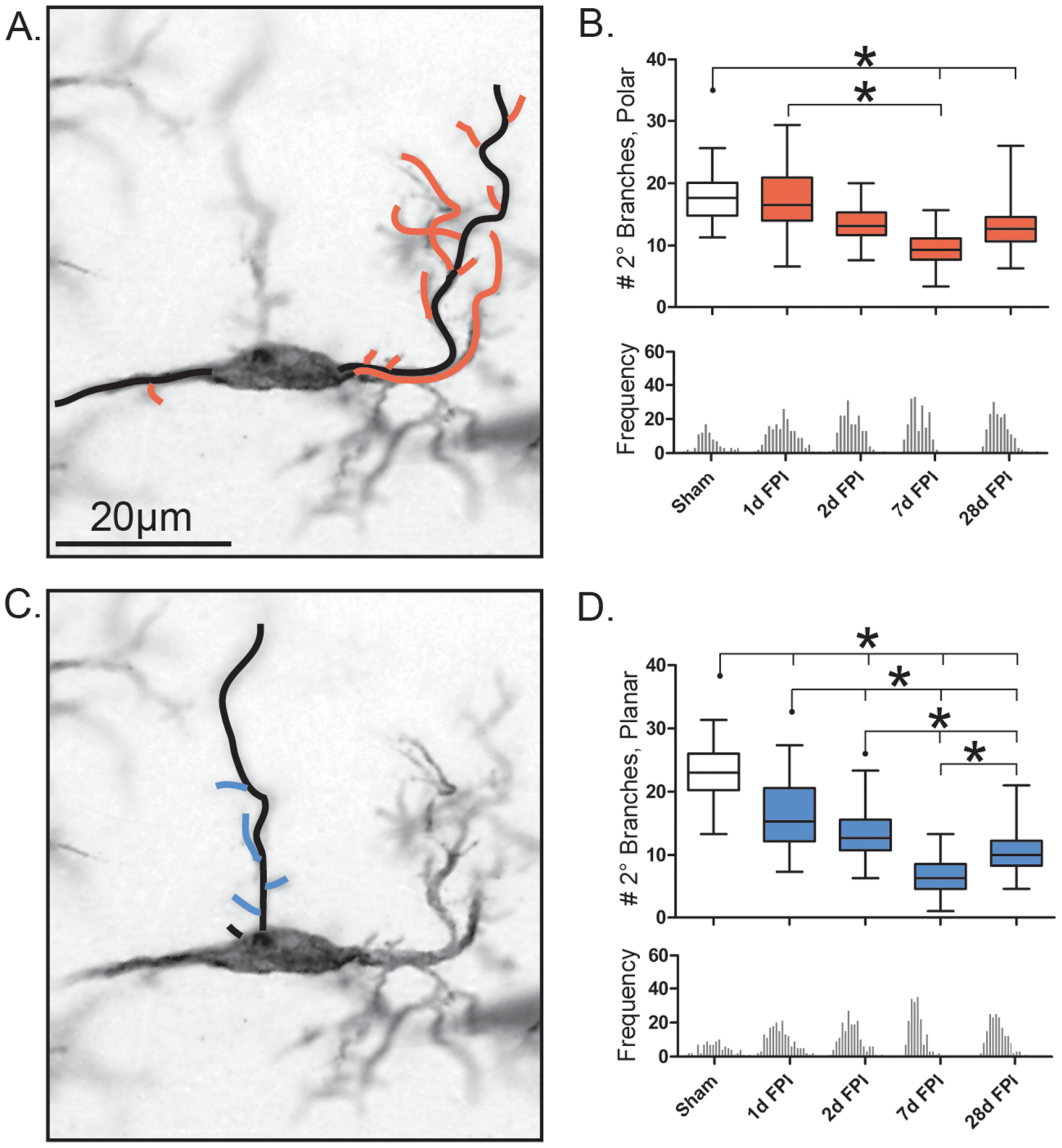
Decrease in the number of rod microglial secondary branches post-injury. (A, C) Representative 28 day post-FPI Iba1-positive rod microglia, 100x. The black lines illustrate the already measure primary branches. The orange and blue lines illustrate the secondary polar and planar branches respectively, secondary branches defined as those directly protruding off of a primary branch. (B) Number of secondary polar branches present on the cell. (D) Number of secondary planar branches present on the cell. Populations described by a box and whisker plot. Notches of each frequency histogram represent the bin center for that specific time point. In both cases a Gaussian distribution is observed. Significance was calculated using the average mean values for each time point (n = 3/time point; *, P<0.05, one-way ANOVA).

The morphological characteristics observed within the primary branches post-FPI was paralleled with similar changes in regards to the secondary branches. The total number of secondary branches (polar and planar) decreased in a similar pattern seen for the total number of primary branches (F_(4, 14)_ = 52.72; p<0.001; data not shown). The total number of secondary polar branches significantly decreased at 7 days and 28 days post-FPI from that of the uninjured sham (F_(4, 14)_ = 11.50; p = 0.0009; Figure 5B). The total number of secondary planar branches significantly changed with injury (F_(4, 14)_ = 182.5; p<0.0001; Figure 5D), whereby at all time points post-injury significantly fewer secondary planar branches compared to sham animals (p<0.001). This effect was most evident on day 7 post-injury.

The ratio of the number of secondary polar branches∶planar branches was significantly different from sham (H = 68.60, df = 5, p<0.0001; Table 1). These changes were evident at all time points post-injury with 1.4 to 2.5 fold increase in the number of branches when compared to sham (p<0.0001).

## Discussion

In 1899, Nissl depicted an array of morphologically distinct microglial cells associated with neurological disease (reviewed in [23]). Still today, there are no specific antibody markers to distinguish between the activation states of microglia. Microglia continue to be classified by morphological features on histological sections. To date, research has focused on the well-known ramified and activated microglia. However, sparse literature exists on rod microglia which featured prominently in Nissl and his students' works (reviewed in [23], [24]). As little data are available on the morphological attributes of rod microglia, this communication defines five characteristics of these cells in the diffusely injured brain and suggests origins and functions for the rod microglia. Here, we show that morphology of rod microglia changes dynamically after diffuse brain injury, primarily through opposing extension and contraction in processes over time post-injury. These characteristics offer the first comprehensive description of rod microglia after CNS insult using a model of diffuse brain injury.

Rod-shaped microglia in sham-injured rats displayed sausage-like soma, as described originally [5]. The orientation of rod-like cells in sham brain is incidental, without a specific polarization of processes with respect to anatomical structures [13]. The ratio of cell length∶cell width was 1∶1 and the soma was 2.5 times longer than it was wide. Additionally, rod-shaped microglia in sham brains had three quarters of the number of polar branches compared to planar. Following brain injury, there was a robust microglial response in the cortex, as reported consistently in experimental brain injury models [13], [17], [25]–[31]. However, rod microglia oriented perpendicular to the dural surface as early as 1 day post-injury, which serve as a novel feature of diffuse brain injury (see [13], [17], [25]). Typically, rod microglia showed the most dramatic changes in the cell length and number of their processes at day 7 post-injury. Although these cells appeared to polarize and elongate, our measurements indicated that the ratio of soma length∶width remained largely unchanged, with statistical differences occurring at day 7 post-injury, at which time the cell length, primary process length and secondary process number also increased. Indeed at day 7, there was a 3-fold increase in the average cell length∶width ratio. Thus, the formation of rod microglia involves polarization, which includes the retraction of planar processes, while extending dominant polar processes. We propose that the term “rod microglia” be used to describe a morphologically distinct cell, which has a cell length∶width of at least 1.5, regardless of changes in the soma. Additionally, the polarized processes should have a reduced number of secondary branches. Microglia become rod microglia after their planar dimensions retract, giving the appearance of elongation.

Alignment of rod microglia with pyramidal neurons perpendicular to the pial surface has been reported in human conditions, including viral encephalitis, HIV-1, general paralysis of the insane [24] and rodent TBI [13]. Rod microglia likely can only form when tissue is preserved [24]; frank degeneration of brain tissue would preclude rod microglia formation. In both human autopsy and experimental models, when rod microglia are present, they are observed juxtaposed to neuronal elements [13], [24]. We conclude that the evolving formation of rod microglia may take more time than offered by rapidly evolving acute degenerative processes (e.g. focal injury, cerebral ischemia). Therefore, we postulate that in the case of destructive lesions where neuronal architecture is destroyed and is paralleled by concomitant accumulation of infiltrating mononuclear cells, the formation of rod microglia may be overwhelmed by destructive processes.

**Figure 6:**
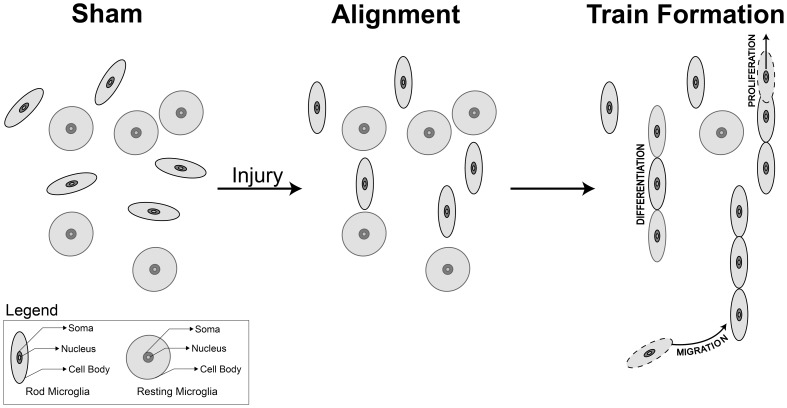
Schematic representation of rod microglia alignment and polarization post-injury. In the sham animals, rod-like microglia are sporadically found throughout all regions of the brain, these cells had sausage-like soma with no defined alignment or polarization in any plane. Post-injury, rod microglia aligned within the S1BF of the cortex, perpendicular to the dural surface. Rod microglia in the diffuse-injured rat brain show a ratio of 1.79±0.03 cell length∶cell width at day 1 post-injury, which increases to 3.35±0.05 at day 7, compared to sham (1.17±0.02). The soma length∶width differs only at day 7 post-injury compared to uninjured sham. Once alignment has occurred, rod microglia form ‘trains’, hypothesized through migration, proliferation and /or differentiation of existing resting microglia. It is clear that cell to cell signals control the migration of microglia, and presumably control their morphology. Microglial function follows morphology, however, the role and signaling cascade for rod microglia has yet to be identified.

Recently, we have shown that rod microglia form trains in the S1BF adjacent to neuronal elements following injury [13] and here, we deepen the characterization of rod microglia cell morphology and propose routes leading to train formation (Figure 6). In the sham-injured animals, rod-like microglia are sporadically found throughout all regions of the brain, with no polarization. Post-injury, rod microglia align within the S1BF of the cortex, perpendicular to the dural surface and alongside neurons. Aligned rod microglia then form ‘trains’, which we hypothesize could occur from migration [32], proliferation [26], [33] and /or differentiation of existing microglia.

Microglia can migrate in response to chemokine signaling originating from glial cells and neurons (reviewed in [34], [35]). Migrating microglia may become rod microglia en route or only undergo transformation upon reaching their chemokine target. Alternatively, microglia may proliferate in response to injury, with daughter cells of microglia becoming either rod or activated morphologies. The cellular factors driving each of these mechanisms are as yet unknown, but likely to involve cell to cell signaling. Future studies aim to determine concrete signals which induce this morphology and pursue them as biomarkers for therapeutic targets. To date, in vitro studies have failed to replicate this in vivo morphology of microglia, likely due to the oversimplification of the complex neuronal-glial signaling required for rod-microglia formation.

The unique morphology of rod microglia permit limited speculation on their role in the injured brain. The fine rods, forming trains adjacent to neuronal elements infer functions that possibly include: splinting damaged neuronal processes, forming a barrier to protect uninjured neurons from an adverse environment, sealing a damaged neuron from further interaction with healthy parenchyma, or simply using neuronal elements to track towards injured areas. In our studies, rod microglia are symmetrical, without a leading or trailing edge, which reduces evidence for simple migration. We continue to explore the hypothesis that rod microglia insulate damaged neurons.

Previous work from our group has investigated the gene expression of M1 and M2 markers in the cortex and thalamus following diffuse brain injury [17]. At 7 and 28 days post-injury, there was evidence of classically activated M1 microglia as well as acquired deactivation. However, there was little evidence for alternatively activated M2 microglia. We apply this evidence to rod microglia to suggest that rod microglia are activated through classical mechanisms, however laser capture microdissection would provide more compelling evidence for a signaling cascade involved in the formation of rod microglia.

Microglia interact with dendrites to form and eliminate dendritic spines [36]. In models of visual deprivation and re-exposure to daylight, microglia change their morphology, appose synaptic clefts more frequently, and envelop synapse-associated elements [4]. To further elucidate the complex organization of these cells, 3D reconstruction could be undertaken. The recently published CLARITY technique, which allows for fully assembled but optically transparent and macromolecule-permeable brain [37], would clearly depict the trajectory and affiliation of rod microglia with neuronal elements in cortical regions with rod microglia. Others have quantified inflammation after acute insult by thresholding digital images without consideration of morphology [38]; these techniques would overlook the distinct involvement of microglial morphologies. Previously, we overcame this technical hurdle by applying the fast Fourier transformation to quantify alignment of microglia after brain injury [39].

The dynamic changes in rod microglia following diffuse brain injury occur as early as day 1 post-injury. The morphological features of injury-induced rod microglia remain evident in cell length and width to day 28 post-injury. The delayed onset and persistent presentation suggest that these cells could play a role in circuit reorganization. Uniquely, diffuse TBI leaves tissue intact (macroscopically), with underlying neuropathology. In our model of diffuse brain injury, general histological stains (e.g. Nissl) give the appearance of healthy tissue [40]. However, this tissue harbors significant neuropathology as indicated by silver accumulation in the S1BF [40], which receives input from thalamic relays connected to whisker stimulation. Indeed, the extent of neuronal activation (cFos staining) in response to whisker stimulation shows hypo-activation followed by hyper-activation in the S1BF over time after experimental diffuse TBI, indicating periods of circuit dismantling followed by reorganization [18]. During the period of reorganization, animals develop behavior changes manifested as sensory sensitivity to whisker stimulation [20], [22]; highlighting the fact that rod microglia may play a role in synaptic plasticity.

Diffuse brain injury is one representative acute neurological injury in which rod microglia play a role. With the new knowledge presented here about rod microglia, the extent to which rod microglia are involved in other neurological diseases can be determined. However, rod microglia appear in experimental diffuse TBI and then behavioral morbidities emerge. The progression of rod microglia and their subsequent decline may occur in other neurological conditions on a time scale unique to each condition. In certain conditions, the disease progression in terms of onset or emergence of behavioral symptoms can outlast rod microglia, such that rod microglia would have waned by autopsy. This may, in part, explain why rod microglia are infrequently found in terminal examination of neurological disease or disease progression. It is tempting to speculate that the close association to neurons by rod microglia leads to synaptic stripping and thus could be related to neurological symptoms unique to each neurological disorder.
